# CD91-Dependent Modulation of Immune Responses by Heat Shock Proteins: A Role in Autoimmunity

**DOI:** 10.1155/2012/863041

**Published:** 2012-11-19

**Authors:** Robert J. Binder, Yu Jerry Zhou, Michelle N. Messmer, Sudesh Pawaria

**Affiliations:** Department of Immunology, University of Pittsburgh, 200 Lothrop Street, Pittsburgh, PA 15261, USA

## Abstract

Heat shock proteins (HSPs) have been known for decades for their ability to protect cells under stressful conditions. In the 1980s a new role was ascribed for several HSPs given their ability to elicit specific immune responses in the setting of cancer and infectious disease. These immune responses have primarily been harnessed for the immunotherapy of cancer in the clinical setting. However, because of the ability of HSPs to prime diverse immune responses, they have also been used for modulation of immune responses during autoimmunity. The apparent dichotomy of immune responses elicited by HSPs is discussed here on a molecular and cellular level. The potential clinical application of HSP-mediated immune responses for therapy of autoimmune diseases is reviewed.

## 1. Introduction: HSPs in Immunity

Expression of HSPs is generally upregulated in cells in response to a variety of stressful conditions including nonphysiological temperature, nutrient deprivation, and hypoxia [1]. It is the inherent chaperoning function of HSPs that allows them to provide their cytoprotective function in assisting correct protein/polypeptide folding and preventing further protein denaturation. It has become evident over the past two decades that the chaperoning function of HSPs also plays a key role in several processes during the development of immune responses [2]. Within the cell, several HSPs act as chaperones of peptides that are ultimately presented by MHC I and MHC II molecules. Thus, the HSPs in the cytosol and in the endoplasmic reticulum form a relay line for the transport of peptides from their formation by the proteasome to the MHC I heavy chain (HC). This is discussed in the next subheading. As the HSPs are some of the most abundant proteins in cells, their liberation into the extracellular environment has been shown to be a key indicator of loss of cellular integrity and they are rapidly recognized by the cellular sentinels of the immune system. Such recognition allows for cross-priming of the potential antigens that the HSPs chaperone. The efficiency of this pathway predicted a cell surface receptor on the cross-presenting cells and that receptor has now been shown to be CD91. These events are discussed in the next two subheadings. The isolation of HSPs (and the associated peptides) from tumor cells or cells infected with pathogens therefore provides a single entity that primes immune responses specific for the chaperoned peptides and thus for the cell that harbored these antigens. This application has been tested in a vast number of rodent models of cancer and infectious disease and is being tested in the clinical setting. We discuss this in the third subheading. A search for optimal immunizing doses of HSPs led to the fortuitous dampening of the immune response at higher doses of HSPs. This phenomenon has been applied to the therapy of autoimmunity and is the focus of the last subheading. This chapter is largely restricted to the HSPs gp96, hsp70, hsp90, and calreticulin although others such as hsp110, grp170 have been shown to elicit similar immune responses [3, 4].

## 2. HSPs Form a Relay Line in MHC I Antigen Processing and Presentation

The classical and current view of antigen processing and presentation by MHC I can be summed up as follows: production of peptides occurs within the cytosol by the large multi-subunit catalytic body called the proteasome. The proteasome ingests polypeptides and trims them down to small peptides usually with the correct C-termini, but extended N-termini, for MHC I binding. The transporter associated with antigen processing (TAP) pumps peptides into the ER in an ATP-dependent manner. Additional N-terminal trimming proteases such as the ER-associated aminopeptidase (ERAP) are present in the ER for final processing [5]. For a period these peptides were envisioned to diffuse to TAP, and once in the ER, they diffuse and get loaded onto MHC HC with the assistance of the peptide loading complex. We now know that diffusion plays a minor role if any at all in antigen presentation; instead, peptide trafficking is possible only because peptides are actually chaperoned by HSPs. This was first proposed in 1994 [6] and the evidence for the involvement of HSPs in peptide processing and presentation is now abundantly clear and comes in both direct and indirect forms. The evidence is as follows: (i) disruption of peptide binding by HSPs abrogates MHC I peptide presentation [7–9], (ii) the extremely poor efficiency of diffusion of peptides within the cell cannot account for the calculated efficiency of antigen presentation [10, 11], unless other more efficient methods of peptide trafficking, such as the HSP chaperoning effect, are integrated into the pathway, (iii) free peptides have not been found in cells even after a careful search for them [12]. The peptides are readily seen once they are released from HSPs by protein denaturation or treatment with ATP or acid [13], (iv) given the hydrophobic residues of amino acids in the hydrophilic environment, solubility issues that this poses [14] must be resolved, and peptide association with HSPs does so, (v) isolation of peptides from highly purified HSP preparations reveals MHC binding peptides and their precursors (intermediates of the processing events) [15–20]; (vi) peptides chaperoned by hsp90 in the cytosol are less processed (longer) than those chaperoned by the ER HSP gp96 revealing a continuum of processing events by proteases in different compartments [15], and (vii) shuttling of MHC binding peptide precursors between HSPs and MHC I HC has been observed in the ER [17]. These lines of experimental evidence suggest and provoke the idea that the relatively recent evolutionary development of the MHC I antigen processing and presentation pathway, a key component of adaptive immunity, has taken advantage of the ancient property of HSP chaperoning. 

## 3. HSP-Peptide Complexes in Antigen Cross-Presentation

The initial event in priming T-cell responses to cancer or infectious disease involves the transfer of antigens from the cells that harbor them to antigen presenting cells. This pathway is called cross-presentation and allows for the presentation of antigens in the form of peptides to T cells in the context of MHC molecules. Cross-presentation of antigens by APCs also directs that the antigen is presented in the context of costimulation, which is a combination of cytokines and a series of APC-T cell interactions through receptors and their corresponding ligands. The specific costimulation received by the naïve T cell dictates the type of T cell response that is primed. HSPs have been shown to play a critical role both in cross-presentation of antigens and in provision of a dynamic set of signals for costimulation. 

Calculations on the amount of antigen that is available for cross-presentation in two independent studies have shown that it is insufficient if the antigens were transferred as a whole protein [21, 22]. This triggered an investigation into the role of HSP-antigen complexes as a necessary alternative to antigen transfer during cross-presentation. Antigens chaperoned by HSPs are cross-presented approximately 50,000 times more efficiently than naked protein and/or peptide alone [22]. This increase of efficiency is in large part due to the presence of the HSP receptor CD91 that is present on APCs [23, 24]. Although the role of HSPs in cross-presentation has been demonstrated *in vivo*, it has been modeled *in vitro* in several antigenic systems in mice and humans over a period of many years (Table 1). These studies have shown that the initial interaction of the APC with the HSP is mediated through the cell surface receptor CD91 and potentially others. The evidence, or lack thereof, for these other suggested HSP receptors is discussed elsewhere [25]. Following binding, the HSP with the chaperoned peptide is internalized into endosomal vesicles. Through an as yet unidentified mechanism, peptides are delivered to the cytosol for trimming by the proteasome where they enter the MHC I processing and presentation pathway. Other mechanisms include internalization of HSP-peptide complexes into MHC I containing vesicles where there can be direct peptide transfer between these molecules [26–30]. However with this mechanism only fully processed peptides chaperoned by HSPs would be presented by MHC I. The requirement for MHC I antigen processing machinery such as the proteasome and TAP appears to be dependent on the antigenic system being tested [7, 24, 31]. Other mechanisms leading to presentation of peptides chaperoned by HSPs may also be dependent on the HSP chaperoning the antigen, as endogenous and exogenous hsp90 have been shown to be directly involved in transendosomal membrane transport [32, 33]. Since the peptides bound to HSPs do not appear to be limited by length or amino acid sequence, HSP chaperoned peptides can also be presented by MHC II of the APC to stimulate CD4^+^ T cells [34–37]. 

By extension of this very efficient mechanism of cross-presentation of HSP-chaperoned antigens, immunization with HSP-antigen complexes primes antigen-specific T-cell responses while comparable amounts of antigen alone does not. This has been demonstrated with gp96 [19, 59–63], hsp90 [15, 62], hsp70 [13, 19, 62], calreticulin [64, 65], hsp110 [3, 4], and grp170 [3, 4]. In these immunization regimens, the HSP-peptide complexes can be purified intact from the antigen bearing cell. Thus, purification of HSPs from tumor cells will yield complexes that represent the entire antigenic fingerprint of that tumor and will prime T-cell responses specific for that tumor. The same applies to cells infected with bacteria or viruses and cells expressing minor histocompatibility antigens or model antigens. The peptides bound to HSPs are not restricted to the MHC haplotype of the originating cells [66]. Various methodologies are also currently available to artificially bind peptides to HSPs noncovalently [67] or covalently through fusion constructs to form immunogenic complexes [68]. In most cases, the immune response measured after priming is of the Th1 phenotype and characterized by CD8^+^ cytotoxic T lymphocytes. In a few situations, immunization with HSPs has led to priming of Th2/antibody or Th17 responses [69–72].

## 4. CD91 Is Pivotal in Regulating HSP-Mediated Costimulation

HSP-chaperoned peptides can be cross-presented by professional APCs; however, presentation and recognition of antigen alone by T cells are not sufficient to prime adaptive immunity. In order to prime T and/or B cells, help is needed from an expanding family of costimulatory molecules on APCs. The cytokine milieu provides additional signals for activation and expansion of these effector cells. The immunogenic HSPs were the first endogenous molecules proven to be particularly adept in stimulating APCs to provide costimulation [73]. Studies have shown that the signals provided by the HSPs to the APCs do not occur through the traditional pattern recognition receptors which include the TLRs. Rather, recent studies have shown that the immunogenic HSPs utilize CD91 to transmit signals to the APC [71]. Primary APCs were shown to be activated by HSPs in a CD91-dependent manner suggesting that CD91 was acting as a signaling receptor for the immunogenic HSPs. The *β*-chain of CD91, which has two NPXY sequences that are consensus motifs for phosphorylation and signal transduction, was subsequently mutated. Upon tyrosine to phenylalanine mutation, CD91 failed to transmit intracellular signals in response to HSP stimulation, abrogating the costimulation provided by the APC. The signaling pathway(s) initiated by CD91 upon HSP stimulation involves the activation of NF-*κ*B and p38 MAPK although other molecules are yet to be identified. Downstream of intracellular signaling, a number of cytokines are released by HSP-stimulated APCs including TNF-*α*, IL-1*β*, IL-6, IL-12, and GM-CSF [71]. Other studies have shown that, in addition to cytokine production by HSP-stimulated APCS, the APCs upregulate expression of costimulatory molecules and maturation markers including CD80, CD86, CD40, and MHC II [74]. The complete array of costimulatory molecules and cytokines is dependent on the type of APC (macrophage or DC subsets) that is stimulated and the HSP (hsp70, hsp90, calreticulin or gp96) that is used for stimulation (Table 2).

CD91 thus has a role in signal 1 (cross-presentation) and 2 (costimulation) that is provided by the APC to T cells in response to extracellular HSP. Similar to HSP-mediated cross-presentation of peptides, other receptors besides CD91 have been suggested to be signaling HSP receptors. However there is abundant published literature that the suggested TLR2/4 receptors were implicated because of the use of endotoxin-contaminated HSP preparations, especially from recombinant sources. A discussion of HSP receptors has been published elsewhere [25]. The flexibility in the pattern of costimulation triggered by various HSPs in a variety of experimental settings has implications in several fields of immunology and we focus here on a discussion on a role in autoimmune diseases.

## 5. Extracellular HSPs and the Etiology of Autoimmunity

As discussed above, APCs stimulated with various HSPs secrete proinflammatory cytokines such as IL-1*β*, IL-6, and TNF-*α* among others (Table 2). In addition, HSPs chaperone self-peptides that can be cross-presented as efficiently as the antigenic peptides. The former of these events has the potential to trigger chronic inflammation during the continuous presence of HSPs in the extracellular environment. The two events concurrently can prime autoreactive T cells if such T cells are not thymically deleted. In at least one autoimmune disease, this concept is strongly supported. The etiology of rheumatoid arthritis remains largely unresolved; however, several factors that *contribute* to the initiation and/or progression of the disease can be pinpointed. The observation of elevated levels of hsp70 in synovial fluids from inflamed joints of RA patients is one of these factors. Hsp70 is found both within the fibroblasts at the joint and in the fluid itself [99, 100]. The significant increase in extracellular hsp70 in arthritic joints is profoundly correlative because nonarthritic joints in the same individual patients have no elevation in hsp70. As mentioned above, hsp70 can interact with its cell surface receptor CD91, and potentially other receptors, on cells to induce the release of proinflammatory cytokines such as IL-1*β*, IL-6, and TNF-*α*. The observation of elevated hsp70 levels in synovial fluids from inflamed joints implicates hsp70 as an initiator of inflammation and/or a perpetrator of these events. In this disease hsp70 will chaperone self-peptides that can be cross-presented by local APCs [101]. Such cross-presentation of self-antigens appears to be sufficient to break tolerance and for priming self-antigen-specific T cells which could contribute to cellular destruction observed in arthritic joints. 

## 6. HSP Immunotherapy for Autoimmune Diseases

Immunization of mice with HSPs typically elicits Th1 responses characterized by CTL specific for antigenic peptides chaperoning the HSP. Optimal immunizing doses range from 1 to 10 *μ*g at the intradermal or subcutaneous route. Upward titrations of this dose revealed a surprising and apparently paradoxical result. Doses of HSPs that were 10 times higher than the immunizing dose administered to mice were shown to prime an immunosuppressive phenotype characterized by expansion of CD4^+^ Tregs [60, 102–104]. The immunosuppressive response could also be transferred to naïve mice by transfer of the expanded CD4^+^ Treg cell population [103]. The phenotype is observed when there is a prior ongoing CTL response, and in at least one system, Tregs from high dose gp96-immunized mice significantly suppressed the IFN-*γ* production by autologous CD4^+^ and CD8^+^ T cells [105]. 

The application of this “high dose” phenomenon has been tested in mouse models of autoimmunity including diabetes and experimental autoimmune encephalomyelitis [103]. In both models, administration of “high doses” of HSPs reduced the severity of disease or prevented its development outright. In the diabetic model, high doses of HSP were administered to NOD mice that were older than 4 months at which point *β*-islet-specific pathogenic T cells were already present in the pancreas. In the EAE model, high dose of HSP was administered after immunizing mice with the MOG peptide which primes pathogenic CD8^+^ T cells. The HSPs used in these studies did not chaperone any antigenic peptides related to the disease model and led to the conclusion that the ability to prime Treg cells was inherent to the HSP molecule itself. The less preferred explanation would be a common self-peptide associated with gp96 regardless of the source of HSP in a global disease setting. Our current understanding of the diversity of responses of APCs stimulated with HSPs (Table 2) sheds some light on the mechanism. It strongly suggests that the disparity of responses in immunization with high and low doses of HSP results from targeting different sets of APCs, possibly through CD91 and additional cell surface receptors, leading to a new repertoire of cytokines and/or costimulatory molecules. Higher doses of HSPs may target different subsets of APCs or stimulate multiple receptors in the same APCs (as the immunogenic dose) to elicit distinct costimulatory profiles [105]. The alternative costimulation could include TGF-*β*, PD-L1, and other Treg skewing molecules and would be predicted to be dominant over other signals. This area is under investigation and definition of these mechanisms will offer novel targets for inhibition in autoimmune diseases.

These data suggest a delicate balance between regulatory and effector T cells mediated by HSPs and is supported by the recent demonstration of significant enhancement of gp96-primed CTL activity after anti-CD25 treatment [106]. By blocking Treg generation, gp96 was able to mediate stronger peptide-specific CTL responses in BALB/c mice and synergistically enhanced gp96 tumor vaccine-induced antitumor immunity.

## 7. Conclusions

Over the past 3 decades the various roles of HSPs in the immune systems have been explored and characterized. It appears that the evolutionarily ancient chaperone functions of HSPs in binding peptides and proteins have been commandeered by the relatively recent development of the adaptive immune system. However recent studies suggest that, parallel to evolution of innate responses, multicellular organisms are alerted to aberrant cellular damage by utilization of pre-existing receptors (CD91) to detect the presence of abundant intracellular molecules (HSPs). We draw many similarities between the innate immune responses elicited by PAMPs through PRRs and those by HSPs through CD91 in terms of costimulation for T-cell priming. Indeed the HSP-CD91 network has been well documented not only in mammals but also in amphibians. While CD91 is a well-studied receptor for HSPs (Table 1), there may be other molecules that may serve as receptors, offering a diversity of responses that may be elicited by each HSP. Again, the diversity of PRRs for recognition of various PAMPs is well noted in innate immunity. The immune responses primed by extracellular HSPs are dictated by the costimulation that is elicited and is as diverse as the APC the HSP will encounter. The immune responses range from antitumor and pathogen immunity to suppressive responses, with the latter being applied to the therapy of autoimmune diseases. With greater understanding of the immunobiology of these proteins, we anticipate that vaccine design will be enhanced.

## Figures and Tables

**Table 1:** A summary of reported antigen cross-presenting systems described for HSP-chaperoned peptides and the corresponding receptor.

HSP	Antigen presenting cell	Uptake mechanism	Reference and year	Notes
rhsp70 (bovine), rhsp70 (human)	Bovine cultured monocytes, B-LCL lines L721.45 and L721.174, and human monocytes	Macropinocytosis	[38] 2010, [39] 2007	CD4 stimulation

rgp96, gp96 and hsp70, rhsp70 (human), hsp70 (human), rhsp70	BMDC, macrophage cell line (p388d1) and DC line (D2SC1), RAW264.7 and RAW309Cr.1, human monocytes, elicited PEC	Receptor (unidentified) mediated endocytosis	[40] 2011, [26] 1999, [41] 2008, [42] 2002, [31] 2000	Differences in lipid raft involvement with N or C terminus of HSP70, also MHC II presentation

gp96, hsp90, hsp70, CRT, (*E. coli* and Mtb) hsp70, gp96 (frog), rhsp70 (human)	B-LCL, human DC, RAW264.7 and peritoneal macrophages, BMDCs, BM-macrophages, CD11c+ cells, frog macrophage-like cells, human PBMC	CD91	[35] 2004, [23] 2000, [24] 2001, [43] 2004, [44] 2004, [45] 2008, [46] 2011	MHC I and II presentation

rhsp70 (human) and rhsp60 (human)	Human monocytes, epidermal LC	CD91	[28] 2002	Internalization with MHC I and II

gp96	Splenic APC, B220+, CD11c+, CD11b+, BMDC	CD91 and partial Lox-1	[37] 2010	MHC II presentation

rhsp70 (human)	Monocytes, MDDC	CD91 and partial CD36/scavenger receptor	[47] 2010	MHC II presentation and CD4 memory

rhsp70 (*M. avium* paratuberculosis)	Bovine macrophage cell line BoMac, bovine DC, macrophages, and monocytes	CD91 and other	[48] 2005	

gp96 (porcine), rgp96 (canine, NTD)	Elicited peritoneal macrophages, MEF-1, PEA-13, DC2.4		[49] 2002, [50] 2010	MHC I and II presentation

hsp90 (human, purified), rhsp72 (human)	RMA-S/A*2402, BMDC	Receptor dependent	[30] 2007	

rhsp90 (human)	BMDC, CHO, HeLa and RAW264.7	SREC-1 and Lox-1	[51] 2010	

rCRT	DC2.4, elicited peritoneal macrophage, MEF-1, PEA-13	SREC-1	[52] 2005	

gp96 (porcine) and rCRT	CHO, elicited peritoneal macrophages, BMDC	SREC-1	[53] 2004	

rhsp70	Human myeloid DC, monocytes, macrophages, CD19+ cells	Lox-1	[54] 2002	

gp96 (porcine) and rCRT	Elicited peritoneal macrophages, RAW264.7, HEK	SRA	[55] 2003	

rgp96 and rCRT	Fibroblasts, BMDC and BM macrophages	SRA and other	[56] 2008	

rgrp170 and rhsp110	RAW264.7, DC1.2, CHO, BMDC	SREC-1 and SRA	[57] 2007	

gp96	D2SC/1, D1, BMDC, splenic DC, macrophages, and B cells	Receptor dependent but not MHC class II or DEC205	[27] 2000	Internalization with MHC I and II

gp96 (porcine)	Elicited peritoneal macrophages, RAW264.7, CHO, COS7, BRL	Unidentified receptor mediated and macropinocytosis	[58] 1999	

gp96 (porcine)	Elicited peritoneal macrophages, RAW264.7, BMDC	Unidentified receptor mediated and macropinocytosis	[29] 2002	

**Table 2:** A summary of reports showing activation of APCs by HSPs.

HSP	Species	Antigen presenting cell	Receptor	Innate immune responses					Reference and year
				Cytokine	Chemokine	NO	Maturation	NF-*κ*B	
hsp60, hsp70	Bacteria, human, mouse	Peritoneal macrophage	ND	IL-12					[75], 1996
hsp60	Human	Macrophage	CD14	TNF-*α*, IL-12, IL-15		Yes			[76], 1999
hsp60	Chlamydia and human	Human EC, SMC, and macrophage	ND	IL-6				Activation	[77], 1999
gp96, hsp70, hsp90	Mouse	DC	ND	TNF-*α*, IL-12, IL-1*β*			↑MHC II, B7-2	Activation	[74], 2000
hsp70	Human	Monocyte	CD14	TNF-*α*, IL-1*β*, IL-6				Activation	[78], 2000
gp96	Mouse	human DC	ND	TNF-*α*, IL-12			↑MHCII, CD86		[79], 2000
hsp60	Mouse	Macrophage	TLR4	TNF-*α*		Yes			[80], 2000
gp96	Mouse	CD11c^+^ cell	ND				↑MHC II, B7-1, B7-2, migration		[81], 2000
hsp70	Mouse	Mouse splenocyte	ND	TNF-*α*, IL-1*β*, IL-6					[82], 2000
hsp70	Mouse	Mouse BMDC	ND	TNF-*α*, IL-12, IL-1*β*, IL-6					[83], 2000
rhsp70	Human	DC	ND				↑CD40, CD86, CD83		[84], 2001
Mtb hsp70	Mycobacteria	THP1, KG1 cells, and DC	CD40		RANTES				[85], 2001
hsp70, gp96	Human	DC	ND				↑HLA-DR, CD40, CD86, CD83, and DC-LAMP		[86], 2001
hsp70	Human	DC	Toll/IL-1R	IL-12					[87], 2002
gp96	Human	BMDC	TLR2/4	IL-12			↑CD86	Activation	[88], 2002
gp96 and hsp70	Human and mouse	Macrophage, immature DC	ND			Yes			[89], 2002
rhsp70	Mycobacteria	Human DC	CD40	IL-12, TNF-*α*	RANTES	Yes	↑CD83, CCR7, CD86, CD80, and MHC II		[90], 2002
hsp60	Human	DC	TLR4	TNF-*α*, IL-12, IL-1*β*					[91], 2003
rhsp70	Human	DC	CD40	IL-12p40					[92], 2003
hsp72	Rat	Splenocyte and macrophage	ND	TNF-*α*, IL-1*β*, IL-6		Yes			[93], 2003
hsp70L1	Human	DC	CD91, TLR2/4	TNF-*α*, IL-12p70, IL-1*β*	IP-10, MIP-1*α*, MIP-1*β*, RANTES				[94], 2004
hsp60	Human	Mouse B-cell	TLR4	IL-10 and IL-6			↑MHC II, CD69, CD40, and B7-2		[95], 2005
HSPB8	Human	DC	TLR4	TNF-*α*, IL-6, IL-12, IL-10			↑CD80, CD83, CD86 and MHC II		[96], 2006
hsp70	Mouse	Tumor cells and DC	TLR4		CXCL10		↑CD80, CD86, CD40, MHC II	Activation	[97], 2009
gp96	Human	Macrophage	TLR2	TNF-*α*, IL-8					[98], 2009
gp96, hsp70 Calreticulin	Mouse	RAW264.7, or PEC	CD91	TNF-*α*, IL-1*β*, IL-6, etc.	CXCL10 (IP-10), CXCL11 (IP-9)			Activation	[71], 2012

ND: not determined; NO: nitric oxide; PEC: peritoneal exudate cells; BMDC: bone-marrow-derived dendritic cells; DC: dendritic cells; EC: endothelial cell; SMC: smooth muscle cell.
